# Two new *Margarinotus* Marseul, 1853 from Jiangxi, Guangxi, and Guangdong provinces (China), with an updated key to all Chinese species (Coleoptera, Histeridae, Histerinae, Histerini)

**DOI:** 10.3897/zookeys.1268.169537

**Published:** 2026-02-06

**Authors:** Jihuan Zheng, Haidong Yang, Tomáš Lackner

**Affiliations:** 1 Guangdong Key Laboratory of Animal Conservation and Resource Utilization, Institute of Zoology, Guangdong Academy of Sciences, Guangzhou, China Institute of Zoology, Guangdong Academy of Sciences Guangzhou China; 2 Department of Environmental Systems Sciences, ETH Zürich, Zürich, Switzerland Department of Environmental Systems Sciences, ETH Zürich Zürich Switzerland

**Keywords:** Coleoptera, Histeridae, Histerinae, Histerini, new species, southern China

## Abstract

Two new species of *Margarinotus* Marseul, 1854 from southern China are described herein: Margarinotus (Grammostethus) mazuri**sp. nov**. and Margarinotus (Ptomister) deficiens**sp. nov**. Subgeneric placement of new taxa is only tentative, pending the phylogeny of the taxon. Almost all specimens were collected using flight interception or pitfall traps. The species Margarinotus (Grammostethus) arrosor (Bickhardt, 1920), **comb. nov**. and Margarinotus (Grammostethus) birmanus Lundgren, 1992 are herein re-described and figured. *Margarinotus arrosor* (Bickhardt, 1920) is transferred into subgenus *Grammostethus* Lewis, 1906 based on the structure of the aedeagus. The taxonomic status of the subgenus *Grammostethus* Lewis, 1906 is briefly discussed, and an updated key to all Chinese species is given. Lectotype and paralectotypes of *Hister gentilis* Lewis, 1891 (= Margarinotus (Grammostethus) birmanus)) are designated.

## Introduction

Histeridae are a moderately sized beetle family of predominantly predaceous taxa distributed all over the world, with the exception of permanently frozen northern lands and polar regions (Kovarik and Caterino 2016). The family is subdivided into nine subfamilies, with the Histerinae being the most species-rich (Mazur 2011; Zhou et al. 2020). Histerinae are further subdivided into five tribes: Exosternini Bickhardt, 1914, Hololeptini Hope, 1840, Platysomatini Bickhardt, 1914, Omalodini Kryzhanovskij, 1972 and Histerini Gyllenhal, 1808. The latter tribe is the most species-rich, with the largest genera: *Margarinotus* Marseul, 1854 (111 valid species), *Hister* Linnaeus, 1758 (191 valid species) and *Atholus* C. Thomson, 1859 (74 valid species), plus several less speciose ones (Mazur 2011). The genus *Margarinotus* was erected by Marseul (1854) with *Hister scaber* Fabricius, 1787, as the type species. According to the recent world catalogue of the Histeridae by Mazur (2011), *Margarinotus* is distributed across Holarctic, Oriental, and Afrotropical regions. Two additional Oriental species: Margarinotus (Promethister) maja Mazur, 2013 (Laos) and Margarinotus (Paralister) koreanus Ôhara, 2016 (Korea) were described after Mazur’s (2011) catalogue, totalling the current 111 valid taxa. The genus is currently morphologically divided into ten subgenera (Mazur 2011): *Margarinotus* Marseul, 1854, *Grammostethus* Lewis, 1906, *Eucalohister* Reitter, 1909, *Paralister* Bickhardt, 1917, *Asterister* Desbordes, 1920, *Ptomister* Houlbert & Monnot, 1923, *Stenister* Reichardt, 1926, *Promethister* Kryzhanovskij, 1966, *Kurilister* Tishechkin, 1992, and *Myrmecohister* Ôhara, 1999. There has never been any attempted phylogenetic reconstruction of the genus, nor the tribe Histerini, and therefore this subgeneric classification is tentative. *Margarinotus* species are generally distinguished from other Histerini genera by the presence of a (complete) outer subhumeral elytral stria, as well as median armature of aedeagus (see Kryzhanovskij and Reichardt 1976 for figures). A key to all Chinese (including Taiwan) Histerini genera, subgenera and species can be found in the recently published *Fauna Sinica* series: Histeroidea, and the reader is referred to the further information and drawings there (Zhou et al. 2022).

The subgenus *Ptomister*, with the largest number of species, was established by Houlbert and Monnot in 1923 as a subgenus of *Hister* Linnaeus. Forty-five species have hitherto been described, including 16 species found in China (Mazur 2011; Zhou et al. 2022). This subgenus can be characterized by the presence of two lateral pronotal striae (marked at least in the anterior pronotal angles, often complete), plus usually complete marginal pronotal stria and non-dilated, asetose tibiae. Furthermore, the pronotal hypomeron is always glabrous and general body form mostly non-cylindrical (Kryzhanovskij and Reichardt 1976).

The subgenus *Grammostethus* was established by Lewis in 1906 as a genus, with *Hister ruficornis* Grimm, 1852 designated as the type species. It can be characterized by rather small body size (PEL: 2.50–4.50 mm), oval, slightly convex black body, pronotum with complete marginal pronotal stria and a single lateral pronotal stria. Furthermore, dorsal elytral striae I–IV are usually present, occasionally stria V is also present, albeit often divided into basal and apical fragments, and the prosternal apophysis often exhibits shortened carinal prosternal striae (Kryzhanovskij and Reichardt 1976). Seventeen species of *Grammostethus* are known, with predominantly oriental distribution, including nine in China (Zhou et al. 2022).

**Figure 1.**
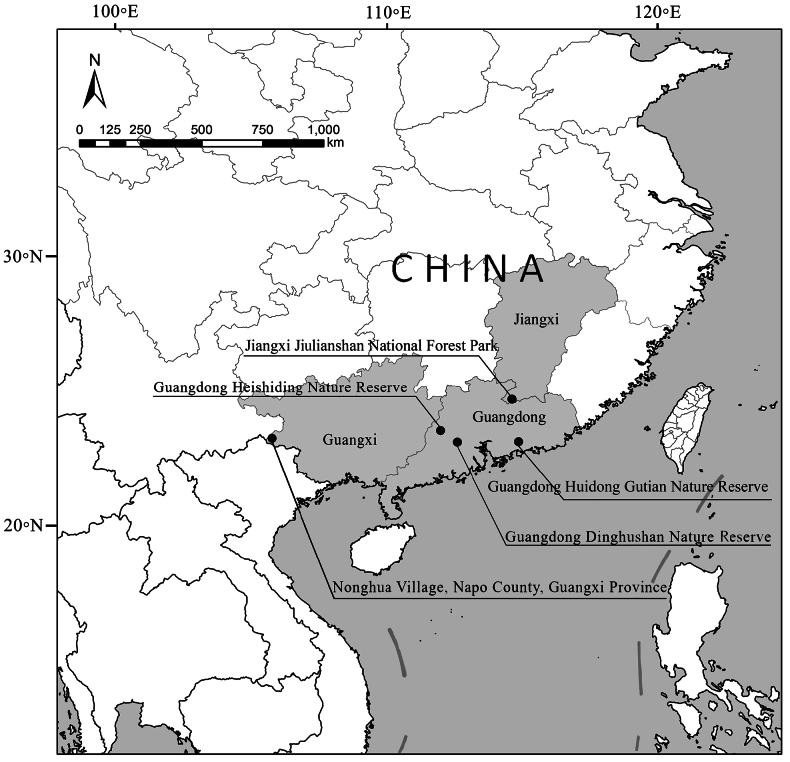
Map of the collecting localities.

From 2020 to 2024, flight interception (FIT) and pitfall traps were used to investigate insect biodiversity in Jiulianshan Natural Forest Park, Jiangxi Province, Gutian Nature Reserve and Dinghushan Nature Reserve, Guangdong Province, southern China (Fig. 1). The obtained Histeridae specimens, together with those already deposited at the collection of the Museum of Biology, Sun Yat-sen University (**SYSU**) (collected from Heishiding natural Reserve, Zhaoqing, Guangdong Province) and at the collections of Institute of Zoology, Chinese Academy of Sciences, Beijing (**IZ-CAS**) were investigated, yielding two undescribed *Margarinotus* taxa, belonging to subgenera *Grammostethus* and *Ptomister*, thus increasing thus the number of described *Margarinotus* species worldwide to 113.

## Materials and methods

Specimens collected during 2020–2024 were preserved in 100% ethanol in small vials. Morphological characters were examined with Zeiss stemi305 microscope. Male genitalia were dissected using the following procedure: abdomen was removed from each specimen, boiled in water for 5–10 min, and then transferred to a vial containing 10% KOH solution. The abdomen with the aedeagus was washed in distilled water three or four times and transferred to a cavity slide using fine forceps. The aedeagus was separated from the abdomen using a hooked, fine dissecting needle. The morphological concepts and body measurements used in this study mostly follow Ôhara (1994), the leg terminology follows Lackner (2010). Abbreviations for measurements of body parts are as follows: **PEL** = length between anterior angles of pronotum and apices of elytra, **APW** = width between anterior angles of pronotum, **PPW** = width between posterior angles of pronotum, **PL** = length of pronotum along mid line, **EL** = length of elytron along sutural line, **EW** = maximal width between outer margins of elytra, **ProW =** maximal width of propygidium, **ProL =** length of propygidium, **PyL =** length of pygidium across median line. Habitus images were taken using a SONY a7 digital camera. Aedeagal images were taken using a Nikon D610 digital camera, attached to a Zeiss V/A1 microscope (with 5× objective lens). A cable shutter release was used to prevent the camera from vibrating. All images were stacked using Helicon Focus 7 to obtain the full depth of focus, and the resulting output was edited with Adobe Photoshop 2025. Type specimens of the species described herein are deposited in the following collections and institutes:

**NHMUK** British Museum of Natural History, London, UK (Max Barclay),

**GIZ** Institute of Zoology, Guangdong Academy of Sciences, Guangzhou, China (J. Zheng),

**IZ-CAS** Institute of Zoology, Chinese Academy of Sciences, Beijing, China (T.-H. Luo),

**CJZH** Jihuan Zheng private collection, Guangzhou, China,

**NMB** Natural History Museum Basel, Basel, Switzerland (M. Borer),

**NMPC** National Museum of Prague Collection, Prague, Czech Republic (L. Sekerka),

**SYSU** Museum of Biology, Sun Yat-sen University, Guangzhou, China (H. Pang),

**MSNG** Museo di Storia Naturale “Giacomo Doria”, Genova, Italy (M. Tavano),

**CTLA** Tomáš Lackner private collection, Munich, Germany,

**ZMHUB** Zoological Museum of the Humboldt University Berlin, Germany (currently no appointed curator).

## Taxonomy

### Genus *Margarinotus* Marseul, 1854

**Subgenus *Grammostethus* Lewis, 1906**

#### Margarinotus (Grammostethus) mazuri sp. nov.

Taxon classificationAnimaliaColeopteraHisteridae

5FC992A7-2671-58E7-81D1-3D7A8100DDB1

https://zoobank.org/E4973DB1-DBA1-437C-A1F1-BC9D7D9A75CF

Figs 2, 3, 4, 5

##### Type material.

***Holotype***: China • ♂, glued on a card, with genitalia in a separate microvial, with following labels: “广东古田保护区 E114°48'28.68", N23°7'19.91", 17-V-2020, FIT, 采集人:李志刚” [China, Guangdong, Huidong, Gutian Nature Reserve, 114°48.48'E, N23°07.33', 17 May 2020, FIT, Zhigang LI leg, printed], followed by: “HOLOTYPE” [red label, printed] (deposited in GIZ). ***Paratypes***: China • 1♂1♀, in alcohol, with following labels: “广东古田保护区, E114°48'34.86", N23°7'16.32", 11-V-2020. FIT, 采集人:李志刚” [China, Guangdong, Huidong, Gutian Nature Reserve, E114°48.58', N23°07.27', 11 May 2020. by FIT(= Flight interception trap), Zhigang LI leg, printed], followed by: “PARATYPE” [yellow label, printed] (GIZ). China • 2 specimens, in alcohol, with following labels: “广东古田保护区, E114°48'38.18", N23°7'21.15", 11-V-2020. FIT, 采集人:李志刚” [China, Guangdong, Huidong, Gutian Nature Reserve, E114°48.63', N23°07.35', 11 May 2020. by FIT(= Flight interception trap), Zhigang LI leg, printed], followed by: “PARATYPE” [yellow label, printed], (GIZ). China • 1♂, in alcohol, with following labels: “广东古田保护区(= GTNR), E114°48'23.64", N23°7'3.71", 27-IV-2020. FIT, 采集人:李志刚” [China, Guangdong, Huidong, Gutian Nature Reserve, E114°48.39', N23°07.06', 27 Apr 2020. by FIT(= Flight interception trap), Zhigang LI leg, printed], followed by: “PARATYPE” [yellow label, printed] (GIZ). China • 1 specimen, in alcohol, with following labels: “江西龙南九连山. E114°27'14.44", N24°35'17.60". 25~30-VI-2021. FIT, 采集人:杨海东” [China, Jiangxi, Longnan, Jiulianshan Nature Reserve, E114°27.24', N24°35.29'. 25–30 Jun 2021. by FIT(= Flight interception trap), Haidong YANG leg, printed], followed by: “PARATYPE” [yellow label, printed] (GIZ). China • 1 specimen, in alcohol, with following labels: “江西龙南九连山, E114°27'52.37" N24°32'17.99". 25~30-VI-2021. FIT, 采集人:杨海东” [China, Jiangxi, Longnan, Jiulianshan Nature Reserve, E114°27.87', N24°32.30'. 25–30 Jun 2021. by FIT(= Flight interception trap), Haidong YANG leg, printed], followed by: “PARATYPE” [yellow label, printed] (GIZ). China • 1 specimen, in alcohol, with following labels: “江西龙南九连山(= JLNR), E114°27'58.01", N24°32'14.87", 10~15-VI-2021, FIT, 采集人:杨海东” [China, Jiangxi, Longnan, Jiulianshan Nature Reserve, E114°27.97', N24°32.25'. 10–15 Jun 2021. by FIT(= Flight interception trap), Haidong YANG leg, printed], followed by: “PARATYPE” [yellow label, printed] (GIZ). China • 1♂1♀, in alcohol, with following labels: “广东鼎湖山保护区,112°32'20.47", N23°09'27.91", 2024.X.29-XI.5. AT10. 采集人:杨海东” [China, Guangdong, Dinghushan, Nature Reserve, 112°32.34', N23°09.47', by pitfall trap, from 29 Oct to 5 Nov 2024, Haidong YANG leg, printed], followed by: “PARATYPE” [yellow label, printed], (GIZ). Three paratypes are deposited in CJHZ and five paratypes are deposited CTLA. 1 female, side-mounted, with following labels: “CHINA mer / Hong-Kong / 1993 / G. de ROUGEMONT” (printed), followed by: “Margarinotus / arrosor / det. S. Mazur” (printed written), followed by the paratype label (CTLA). 2 exs., “CHINA: Guangdong Prov., / Nanling National Nature Reserve / Dadongshan, 18-21.iv.2013 / 24°55.92'N, 112°42.99'E, 730m / J. Hájek & J. Růžička leg.” [printed], followed by: “baited pitfall trap (fish meat) / secondary vegetation with shrubs / and trees on steep slope along / path to waterfall in small valley / behind headquarters, cold and / rainy weather” [printed], followed by the paratype label (NMPC).

##### Differential diagnosis.

By the presence of both inner-and-outer lateral pronotal striae the newly described species should key out to the subgenus *Ptomister* (using various subgeneric keys, e.g. Zhou et al. 2022 or Kryzhanovskij and Reichardt 1976). However, the presence of apically “open” un-fused parameres with eversible median lobe classifies this taxon into subgenus *Grammostethus* (see also discussion below). *Margarinotus* (*G.*) mazuri sp. nov. and M. (G.) arrosor (Bickhardt, 1920), comb. nov. differ from the remaining Chinese *Grammostethus* by the presence of both complete lateral pronotal striae. From M. (G.) arrosor it differs by the more slender body shape, lighter cuticle, complete four dorsal elytral striae, much denser propygidial and pygidial punctation, shorter and stouter aedeagus with a characteristic translucent ring near phallobase (this condition is shared with M. (G.) birmanus, see also below).

##### Description.

Body length: 4.08–5.39 mm (PEL: average = 4.48, *n* = 11); width, 3.35–4.37 mm (EW: average = 3.64, *n* = 11). Biometric data are given in Table 1. Body oblong-oval, black, shiny; legs and antennae reddish brown.

**Table 1.** Biometric data of the newly described *Margarinotus* species.

	M. (G.) mazuri (*n* = 11)	M. (P.) deficiens (*n* = 5)
PEL	4.48 (4.08–5.39)	5.92 (5.42–6.31)
APW	1.52 (1.32–1.72)	2.08 (1.98–2.14)
PPW	3.00 (2.66–3.34)	4.14 (3.95–4.46)
EL	2.68 (2.54–3.31)	3.54 (3.29–3.77)
EW	3.64 (3.35–4.37)	4.82 (4.32–5.16)
PL	1.85 (1.59–2.31)	2.56 (2.41–2.84)
ProW	2.37 (2.55–2.05)	2.85 (2.56–3.05)
ProL	1.16 (0.93–1.31)	1.19 (0.95–1.38)
PyL	1.44 (1.29–1.56)	1.55 (1.48–1.74)

**Figure 2.**
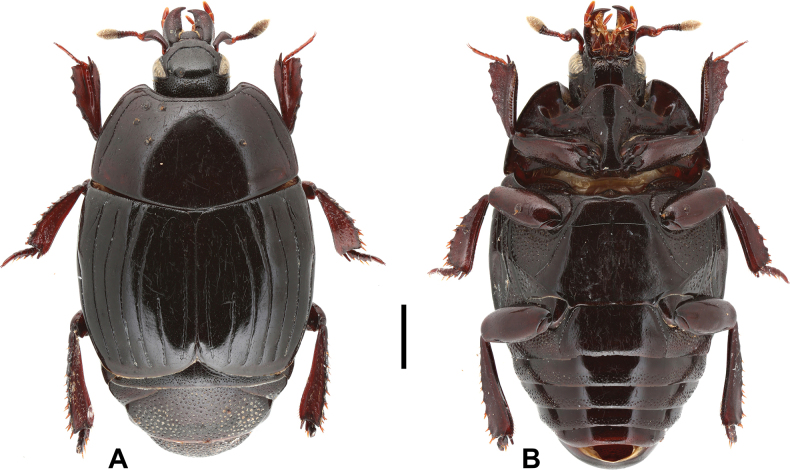
Margarinotus (Grammostethus) mazuri sp. nov., habitus. **A**. Dorsal view; **B**. Ventral view. Scale bar: 1.0 mm.

***Head***: frontal stria (Fig. 2) complete, deeply impressed, anteriorly straight. Mandibles stout; sub-apical tooth short; labrum semi-circular anteriorly. Frons, epistoma and dorsal surface of mandibles consistently and wholly covered with fine punctures.

***Pronotum***: marginal pronotal stria complete laterally, broadly interrupted behind head (Fig. 2A). Outer lateral pronotal stria complete, occasionally slightly shortened basally. Inner lateral pronotal stria complete and strongly crenate, slightly bisinuate medially. Disk of pronotum clothed with sparse microscopic punctures; hypomeron glabrous.

***Elytra***: disk sparsely clothed with microscopic punctures; elytral humeri inconspicuous. Elytral epipleuron densely and coarsely punctate; marginal elytral stria absent basally, present on apical half; marginal epipleural stria complete, in dense punctures. External subhumeral, as well as dorsal elytral striae I–IV complete, stopping short of elytral apex. Fifth dorsal elytral stria slightly shorter than sutural one, with short basal rudiment. Sutural elytral stria present on apical two-thirds, basally abbreviated. Oblique humeral stria present on basal third. All striae, except for oblique humeral one, strongly crenate.

**Figure 3.**
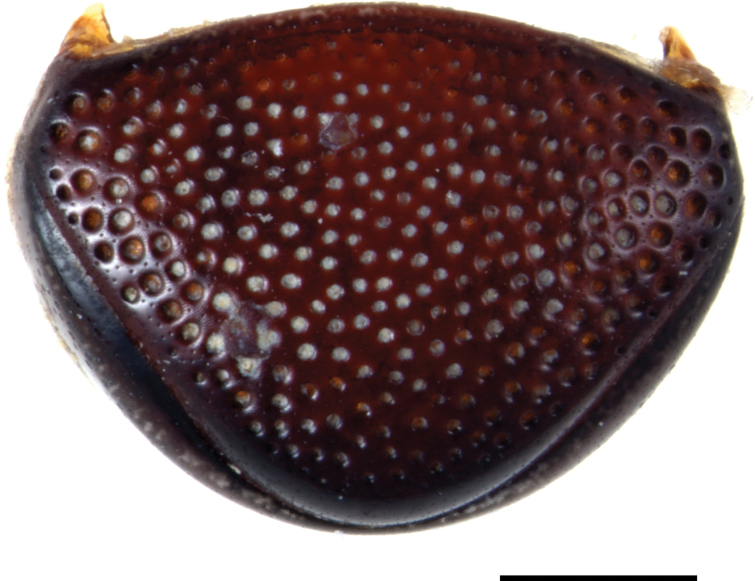
Margarinotus (Grammostethus) mazuri sp. nov., pygidium. Scale bar: 0.5 mm.

***Abdomen***: pygidium (Fig. 3) and propygidium with dense and deep punctures, separated by 0.5–1.5 × their diameter, growing in size laterally, intermingled with minute punctures. Punctures of pygidium denser than those of propygidium.

**Figure 4.**
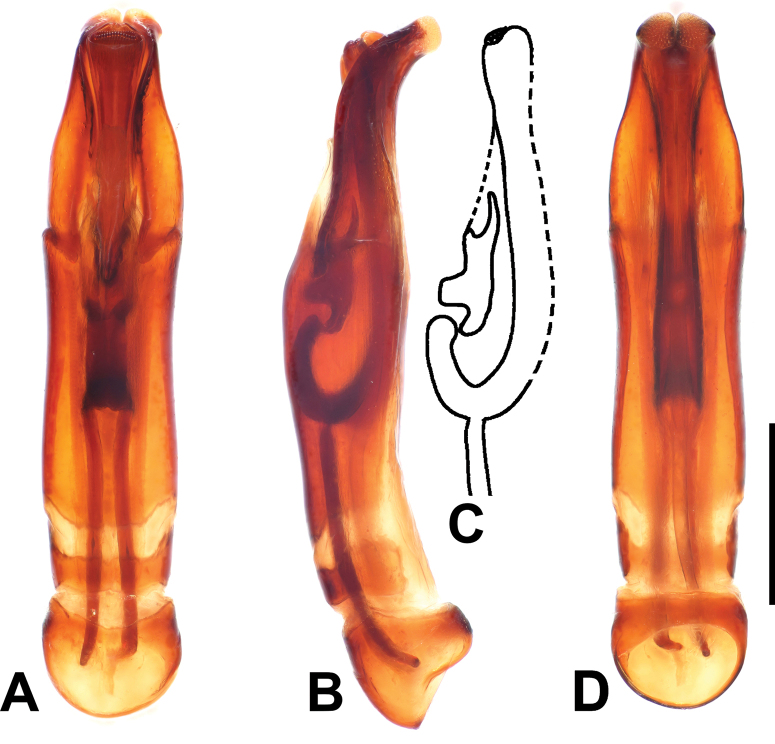
Margarinotus (Grammostethus) mazuri sp. nov., aedeagus. **A**. Dorsal view; **B**. Ditto, lateral view; **C**. Median armature; **D**. Aedeagus, ventral view. Scale bar: 0.5 mm.

**Figure 5.**
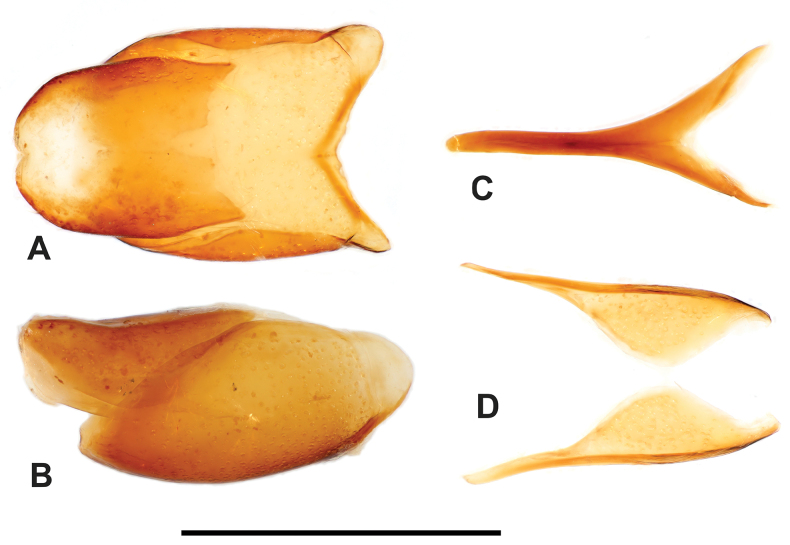
Margarinotus (Grammostethus) mazuri sp. nov., male terminalia. **A**. 8^th^ tergite and sternite, ventral view; **B**. Ditto, lateral view; **C**. 9^th^ sternite, ventral view; **D**. 9^th^ and 10^th^ tergites, ventral view. Scale bar: 1.0 mm.

***Prosternum***: prosternal lobe rounded anteriorly; marginal prosternal stria complete, at times medially narrowly interrupted, well impressed and subcariniform laterally. Disk of prosternal lobe coarsely punctate laterally, punctures becoming finer and sparser medially. Carinal prosternal striae rudimentary, present as short fragments on prosternal apophysis, occasionally absent; lateral prosternal striae carinate, bisinuate, curved inwardly apically.

***Mesoventrite and metaventrite***: anterior margin of mesoventrite feebly emarginate medially; marginal mesoventral stria complete, carinate (Fig. 2B). Disk of mesoventrite almost glabrous, sparsely clothed with fine punctures; meso-metaventral suture complete, finely impressed. Lateral metaventral stria (Fig. 2B) extending obliquely and posteriorly, bent inwardly at apical end, not united with oblique stria that extends inwardly from middle of metaventral-metepisternal suture; intercoxal disk of metaventrite sparsely clothed with fine punctures throughout, several coarse punctures present along lateral metaventral stria. Lateral disk of metaventrite on basal half densely covered with large setiferous punctures, becoming smaller on posterior half, intermingled with microscopic punctures. Intercoxal disk of first visible abdominal sternite completely striate laterally, densely and moderately punctate laterally, medially glabrous.

***Legs***: protibia with six rather blunt teeth topped by small denticle on outer margin, diminishing in size in proximal direction; protarsal groove deep, straight. Protibial spur stout, conspicuous. Mesotibia on outer margin with a row of short denticles, becoming doubled and growing in size distally. Mesotibial tarsal claws very short, their length ~ 1/3 of ultimate mesotarsomere’s length. Metatibia in all aspects similar to mesotibia, albeit more slender; claws of ultimate metatarsomere ~ 1/2 its length.

##### Biology.

Unknown, collected by pitfall as well as flight interception traps.

##### Distribution.

China (Guangdong, Jiangxi, Hong-Kong).

##### Etymology.

Named after our dear colleague and friend, excellent Polish specialist of Histeridae, Prof. Sławomir Mazur (Warsaw, Poland).

##### Remarks.

No variation in elytral dorsal stria configuration was observed. Two specimens collected from Dinghushan Nature Reserve by pitfall traps exhibit an interrupted outer lateral pronotal stria.

#### Margarinotus (Grammostethus) arrosor

Taxon classificationAnimaliaColeopteraHisteridae

(Bickhardt, 1920) comb. nov.

D47A0A27-2829-5ED7-B130-B62094A3B0E9

Figs 6, 7, 8, 9

Hister arrosor Bickhardt, 1920: 98, 99 (description, key).
Margarinotus arrosor : Wenzel 1944: 126, 132 (catalogued, key).
Margarinotus (Ptomister) arrosor : Kryzhanovskij and Reichardt 1976: 332, 334 (key, re-description). Mazur 1984: 164 (catalogued). Mazur 1997: 92 (catalogued). Mazur 2011: 78 (catalogued). Lackner et al. 2015: 100 (catalogued).

##### Material examined.

China • 1 ♂, glued on a card, with genitalia in a separate microvial, with following labels: “江西龙南九连山, E114°27'52.37" N24°32'17.99", 5-10.VI.2021, FIT, 采集人:杨海东” [= China, Jiangxi, Longnan, Jiulianshan Natural Park, E114°27.87', N24°32.30'. 5–10 Jun 2021. By FIT(= Flight interception trap), Haidong YANG leg.] (GIZ). China • 1♂, in alcohol, with following labels: “广东古田保护区,17.V.2020. 采集人:李志刚” [= China, Guangdong, Huidong, Gutian Nature Reserve, 17 May 2020. By FIT(= Flight interception trap), Zhigang LI leg]. (GIZ). China • 1♀, in alcohol, with following labels: “广东古田保护区, 28.IV.2021. FIT 采集人:李志刚” [= China, Guangdong, Huidong, Gutian Nature Reserve, 28 Apr 2021. By FIT(= Flight interception trap), Zhigang LI leg.]. (GIZ). China • 1 specimen, glued on a card, with following labels: “广东鼎湖山保护区 E112°32'53.19" N23°9'28.55", 19-24.IV.2021. FIT. 采集人:杨海东” [= China, Guangdong, Zhaoqing, Dinghushan Nature Reserve, E112°32.89' N23°09.48', 19–24 Apr 2021. By FIT(= Flight interception trap), Haidong YANG leg.] (GIZ). China • 1 specimen, glued on a card, with following labels: “广东鼎湖山保护区 E112°32'34.36" N23°10'18.01", 28.IV-1.V.2021, FIT, 采集人:杨海东” [= China, Guangdong, Zhaoqing, Dinghushan Nature Reserve, E112°32.57' N23°10.30', from 28 Apr to 1 May 2021. By FIT(= Flight interception trap), Haidong YANG leg.] (CJZH). 1 ex., “China Jiangxi Prov. [NF 10] / Jinggangshan Mts., Songnuping / 26°34.7'N, 114°04.3'E, 1280 m / (stream valley), 27.iv.2011 / Fikáček, Hájek, Jia & Song”, “cut and decaying tops of / bamboo trunks in sparse / bamboo bush” (NMPC). 1 ex., “China: Zhejiang Prov., Lin’an Co. / West Tianmushan Nat. Res. / 0.5 km W Orig.Temple of Lion / Sect, 30.3415°N 119.4279°E, / J. Hájek & J. Růžička leg.”, “2.–6.vii.2017, 1200m/ baited pitfall traps #07 (fish meat / ripening cheese), broad-leaved / deciduous forest, junction of Blind / Alley and track to immortal Peak” (NMPC).

##### Differential diagnosis.

By both lateral pronotal striae being complete, M. (G.) arrosor comb. nov. is similar to M. (Grammostethus) mazuri sp. nov., differing from it by a more circular body shape, darker cuticular colour, generally larger body size, only three complete parallel dorsal elytral striae (M. (G.) mazuri has four complete striae), less densely punctate propygidium and pygidium and more slender aedeagus devoid of translucent basal ring (present in M. (Grammostethus) mazuri). The anterior margin of the mesoventrite of M. (G.) arrosor comb. nov. is almost straight, feebly emarginate medially, similar to M. (Grammostethus) mazuri.

##### Re-description.

Body length: 4.51–5.58 mm (PEL: average = 5.03, *n* = 5); width, 3.88–4.85 (EW: average = 4.32, *n* = 5). Body oval, black, shiny; body appendages castaneous.

***Head***: frontal and supraorbital striae complete, straight anteriorly. Mandibles sparsely and finely punctate; frontal disk, clypeus, and labrum with microscopic punctures.

**Figure 6.**
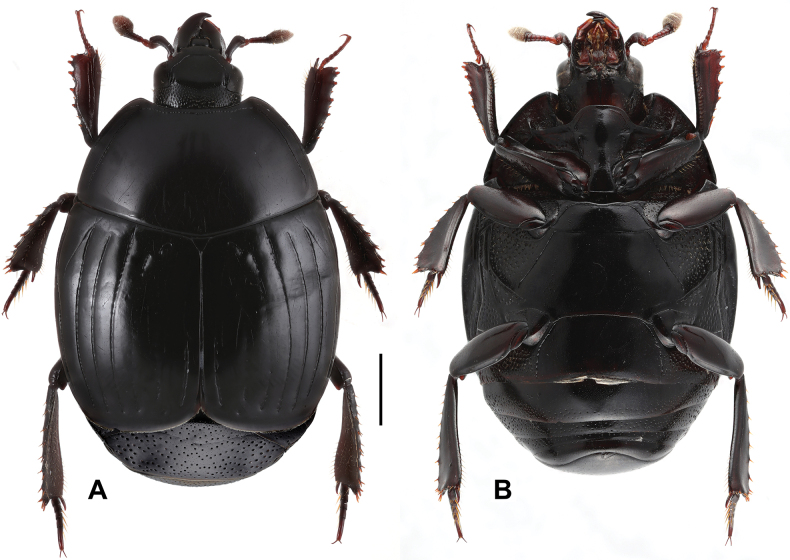
Margarinotus (Grammostethus) arrosor comb. nov., habitus. **A**. Dorsal view; **B**. Ditto, ventral view. Scale bar: 1.0 mm.

***Pronotum***: marginal pronotal stria complete laterally, broadly interrupted behind head (Fig. 6); outer lateral stria complete; inner lateral stria complete, strongly crenate, slightly bisinuate medially, almost parallel to outer lateral stria. Disk of pronotum clothed with microscopic punctures, almost glabrous. Pronotal hypomeron glabrous.

***Elytra***: elytral disk clothed with microscopic punctures, almost glabrous; elytral epipleuron densely and coarsely punctate. Elytral humeri inconspicuous. Marginal elytral stria absent basally, present on apical elytral half; marginal epipleural stria complete, in dense punctures. External subhumeral as well as dorsal elytral striae I–III complete; dorsal elytral IV stria intermittent, medially absent, present as basal and apical fragments. Dorsal elytral striae I–III very regular, parallel-sided; stria V present as basal and apical rudiments; sutural elytral stria present on apical two-thirds. Oblique humeral stria finely impressed, present on basal third. All striae, except the oblique humeral one, deeply impressed but impunctate.

**Figure 7.**
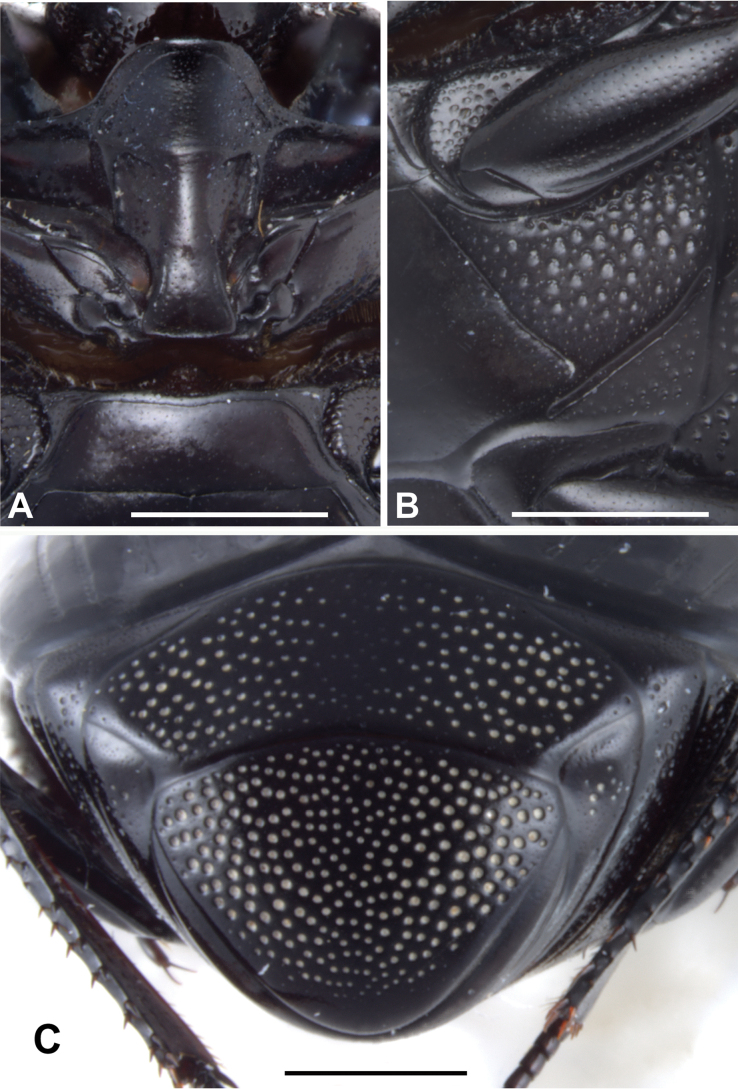
Margarinotus (Grammostethus) arrosor comb. nov. **A**. Prosternal process and mesoventrite; **B**. Intercoxal and lateral disks of metaventrite; **C**. Propygidium+pygidium. Scale bar: 1.0 mm.

***Abdomen***: pygidium and propygidium with an indistinct depression postero-laterally, with variously sized sparse punctures, intermingled with minute punctures. Propygidial punctures increasing in size and density laterally (Fig. 7). Punctures of pygidium denser and more regular than those of propygidium; pygidial apex glabrous.

***Prosternum***: prosternal lobe anteriorly rounded; marginal prosternal stria complete, well impressed, laterally subcariniform, interrupted medially. Disk of prosternal lobe coarsely punctate laterally; punctures becoming finer and sparser medially. Carinal prosternal striae present as short fragments on prosternal apophysis (Fig. 7); lateral prosternal striae similar to those of preceding species.

***Mesoventrite***: anterior margin of mesoventrite almost straight, feebly emarginate medially; marginal mesoventral stria complete, crenulate. Mesoventral disk sparsely clothed with fine punctures, almost glabrous. Meso-metaventral suture complete, slightly bisinuate.

***Metaventrite***: lateral metaventral stria (Fig. 7) fine, almost straight, not united with oblique stria that extends inwardly form middle of metaventral-metepisternal suture (Fig. 7). Intercoxal disk of metaventrite sparsely clothed with fine punctures throughout, almost glabrous, a few coarse punctures present along lateral metaventral stria. Lateral metaventral disk densely covered with large setiferous punctures basally, becoming smaller posteriorly, intermingled with fine minute punctures. Intercoxal disk of first visible abdominal sternite completely striate laterally, glabrous.

***Legs***: protibia with seven moderately large teeth on outer margin, topped by small denticle; protibial groove deep, straight; protibial spur stout, long; outer face of protibia finely punctate and variolate. Mesotibia with a row of rather short denticles on outer margin, becoming doubled and slightly growing in size distally; mesotarsal claws of ultimate mesotarsomere ~ 1/2 its length. Metatibia in all aspects similar to mesotibia, but more slender and denticles on outer margin shorter.

**Figure 8.**
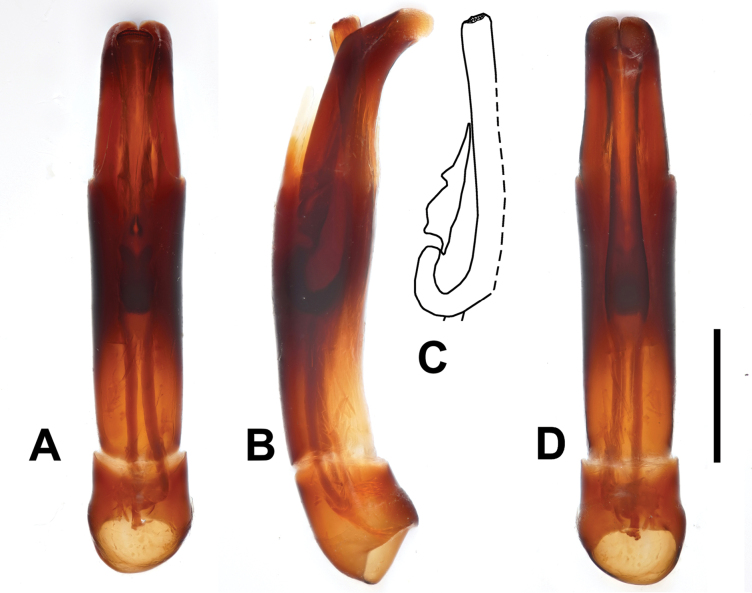
Margarinotus (Grammostethus) arrosor comb. nov., aedeagus. **A**. Dorsal view; **B**. Ditto, lateral view; **C**. Median armature; **D**. Aedeagus, ventral view. Scale bar: 0.5 mm.

**Figure 9.**
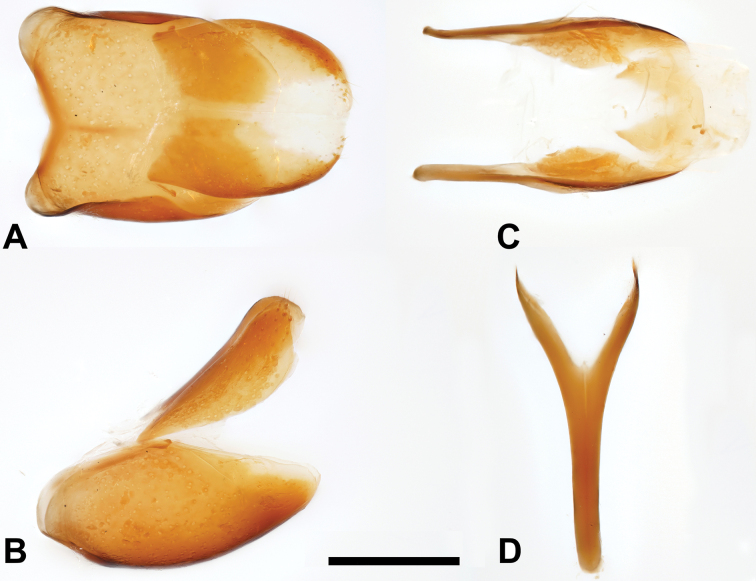
Margarinotus (Grammostethus) arrosor comb. nov., male terminalia. **A**. 8^th^ tergite and sternite, ventral view; **B**. Ditto, lateral view; **C**. 9^th^ and 10^th^ tergites, dorsal view; **D**. 9^th^ sternite, ventral view. Scale bar: 0.5 mm.

***Male genitalia***: aedeagus: basal piece short; ratio to parameres 1:5.5. Tegmen tubular, similar to the preceding species, but narrower; parameres narrowing apically (Fig. 8). Tergites and sternites VIII–X as in Fig. 9.

##### Distribution.

Originally described from China (Fujian), new to provinces Guangdong, Zhejiang, and Jiangxi.

##### Biology.

Unknown, collected by flight interception traps.

##### Remarks.

Bickhardt (1920) described *Hister arrosor* based on two specimens: one from “China” (kept in his private collection, housed now in ZMHUB) and another one from “Prov. Fo-kien” [= Fujian Prov., China], housed in Hamburg Museum of Natural History, Germany. Since the collections of Hamburg museum were destroyed during WWII (see also Lackner 2015: 96) it is possible that only one syntype housed in ZMHUB remains. Unfortunately the Coleoptera section of the entomological collections at ZMHUB has been closed temporarily due to the absence of curators. We have therefore not been able to compare our specimens with the type and identified them only by careful comparison with Bickhardt’s description. The species is herein figured for the first time.

*Margarinotus* (*G.*) *arrosor*, like M. (G.) mazuri sp. nov. possesses double lateral pronotal striae, which hitherto classified it to subgenus *Ptomister* by all previous authors. However, due to the apically “open” parameres and eversible median lobe it is herein transferred into *Grammostethus* (see also discussion below).

During the sampling by the flight interception (FIT) method Margarinotus (Grammostethus) arrosor comb. nov. has been observed to be much less common than its above-described congener M. (Grammostethus) mazuri sp. nov.

#### Margarinotus (Grammostethus) birmanus

Taxon classificationAnimaliaColeopteraHisteridae

Lundgren, 1992

B786E5EA-6DB3-5B7D-A386-C9878806F36E

Figs 10, 11, 12

Hister gentilis Lewis, 1891: 25 (not Horn, 1883: 285). Lundgren in Johnson et al. 1992: 12.
Grammostethus gentilis Lewis, 1906: 400 (included in Grammostethus).
Margarinotus gentilis Wenzel, 1944: 26 (catalogued).
Margarinotus (Grammostethus) gentilis : Mazur 1984: 175 (catalogued).
Margarinotus (Grammostethus) birmanus : Mazur 1997: 100 (catalogued). Mazur 2007: 75 (distribution). Mazur 2011: 83 (catalogued). Gomy and Vienna 2013: 160 (distribution). Mazur 2013: 190 (distribution). Lackner et al. 2015: 99 (catalogued).

##### Type material examined.

***Lectotype*** (present designation), male, side-mounted on a mounting card, genitalia extracted and glued to the same mounting card as specimen, with following labels: “Carin Chebá / 900-1100 m / I. Fea V XII-88” (black-framed, printed label), followed by: “gentilis / Lewis” (black-framed, written label), followed by: “Typus” (red-framed, red-printed label), followed by: “Hister / gentilis / Lewis” (written), followed by: “Museo Civico / di Genova” (light green, black-framed printed label), followed by: “SYNTYPUS / Hister / gentilis / Lewis, 1891” (light-red, printed-written label), followed by: “LECTOTYPE / *Hister gentilis* / Lewis, 1892 / Des.T. Lackner 2025” (red label, printed). ***Paralectotypes***: 2 females+1 male, with following labels: “Carin Chebá / 900-1100 m / I. Fea V XII-88” (black-framed, printed label), followed by: “SYNTYPUS / Hister / gentilis / Lewis, 1891” (light-red, printed-written label), followed by: “Museo Civico / di Genova” (light green, black-framed printed label), followed by: “Paralectotype / *Hister gentilis* / Lewis, 1892 / Des.T. Lackner 2025” (red label, printed) (MSNG). Paralectotype, sex undetermined, genitalia extracted, but not present with the specimen (probably a male), side-mounted on a triangular mounting card, both protarsi as well as right mesotarsus missing, with following labels: “Carin Chebà / 900-1000 m. / L. Fea V XIII-88” (printed, black-margined label), followed by: “Hister / gentilis / Type. Lewis” (written), followed by: “Museo Civ / Genova” (orange label, printed), followed by: “SYN- / TYPE” (blue-margined, round label, printed), followed by: “G. Lewis Coll. / B.M. 1926-39” (printed), followed by: “qr code / NMHUK 014436786” (printed), followed by: “Paralectotype / *Hister gentilis* / Lewis, 1892 / Des.T. Lackner 2025” (red label, printed) (NHMUK). Paralectotype, female, side-mounted on a triangular mounting card, with following labels: “Carin Chebà / 900-1000 m. / L. Fea V XIII-88” (printed, black-margined label), followed by: “Hister / gentilis” (written), followed by: “Museo Civ / Genova” (orange label, printed), followed by: “SYN- / TYPE” (blue-margined, round label, printed), followed by: “G. Lewis Coll. / B.M. 1926-39” (printed), followed by: “qr code / NMHUK 014436787” (printed), followed by: “Paralectotype / *Hister gentilis* / Lewis, 1892 / Des.T. Lackner 2025” (red label, printed). (NHMUK).

**Figure 10.**
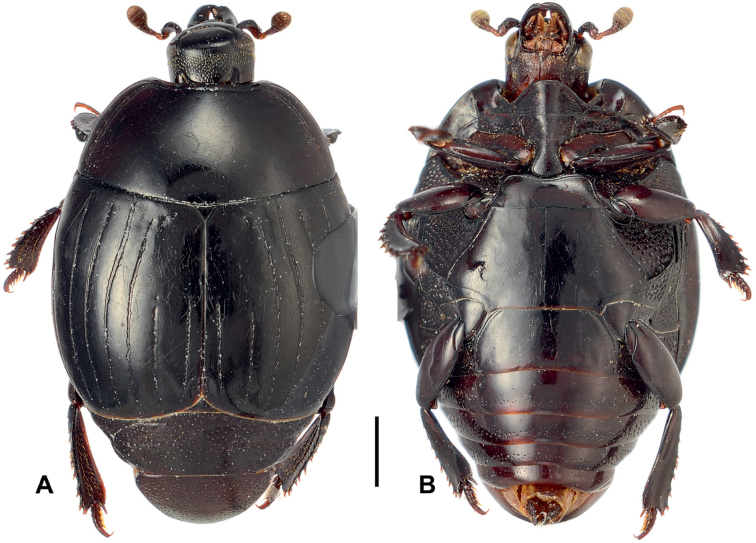
Margarinotus (Grammostethus) birmanus Lundgren, 1992 (= *Hister gentilis* Lewis, 1891), paralectotype, habitus. **A**. Dorsal view; **B**. Ventral view. Scale bar: 1.0 mm.

**Figure 11.**
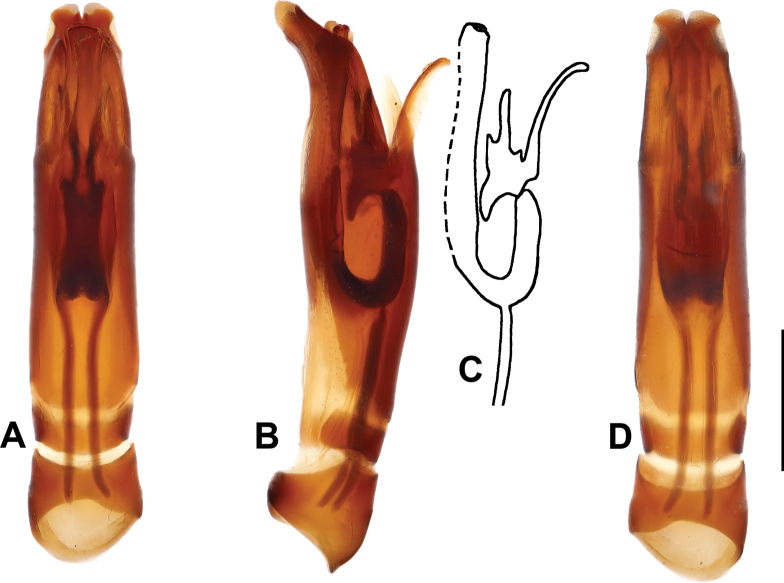
Margarinotus (Grammostethus) birmanus Lundgren, 1992 (= *Hister gentilis* Lewis, 1891), paralectotype, aedeagus. **A**. Dorsal view; **B**. Ditto, lateral view; **C**. Median armature; **D**. Aedeagus, ventral view. Scale bar: 0.5 mm.

##### Additional material examined.

Laos. 1 spec., “LAO-NE, Hua Phan / prov., 20°12'N, 104°01'E / PHU PHAN Mt. / ~ 1750m, 17.v.3.vi. / 2007, Vít Kubáň leg.”, “NHMB, / expedition to / Laos, 2007” (NMB), **with doubt**. 1 male + 1 female: “LAOS Houa Phan Prov. / Sam Neua / Ban Saleuy (1200m) / 6.v.2012, P. Guérard leg.” (ZSM). 1 spec., Laos, Bokeo Province, 5 km W of Ban Toup, Bokeo Nature Reserve, 500–700m, 20°27-28'N / 100°45'E, 4.-18.v.2011. NMHB Basel, Laos 2011 Expedition, M. Brancucci, M. Geiser, D. Hauck, Z. Kraus, A. Phantala & E. Vongphachan lgt. (MSNG).

##### Re-description.

***Measurements***: PEL: 5.60–6.00 mm; EL: 3.50–4.00 mm; APW: 2.00–2.20 mm; PPW: 4.20–4.50 mm; EW: 5.10–5.70 mm. Oval, black, shiny; dorsum of elytra and pronotum with scattered microscopic punctures.

***Head***: frontoclypeal region flattened, glabrous; frontal stria angulate, medially straight; supraorbital stria complete; occipital stria absent. Eyes prominent; mandibles smooth, prognathous, elongated; labrum almost semi-circular. Other mouthparts not examined; antennae rather long; club typical for Histerini, with two distinct sutures.

***Pronotum***: lateral sides strongly convergent anteriorly; marginal pronotal stria thin, complete; outer lateral pronotal stria on apical end slightly inwardly bent, shortened basally; inner lateral pronotal stria slightly bisinuate; basal end slightly inwardly bent, otherwise complete laterally and anteriorly; surface ventrad to it with scattered punctures. Pronotal hypomeron glabrous.

***Elytra***: elytral epipleuron with dense deep punctures; marginal epipleural stria fine, complete. Humeral elytral stria fine, short; outer subhumeral stria almost complete, deeply impressed, shortened on both ends. Dorsal elytral striae I–IV almost complete (in case of at least one Laotian specimen stria IV is weakened and intermittent), running sub-parallel; stria V present on apical half (approximately) and as well as short curved basal fragment; sutural elytral stria slightly longer, surpassing elytral half basally.

***Abdomen***: propygidium with faint irregular depressions; punctures deep, separated by ~ 1–2 × their diameter; pygidium with similar punctuation; punctures becoming smaller apically, on apical third punctures very fine, dense, and small.

***Prosternum***: prosternal lobe apically rounded; marginal prosternal stria fine, complete, laterally prosternal lobe with several deep punctures. Pronotal process broad, slightly convex; lateral pronotal striae short, carinate; carinal prosternal striae present between procoxae, basally united; lateral sides of prosternal process with sparse deep punctures.

***Mesoventrite***: glabrous; marginal mesoventral stria complete and carinate.

***Metaventrite***: intercoxal disk glabrous; lateral metaventral stria straight, complete, its apical end attaining oblique metaventral stria; lateral disk of metaventrite with large dense setigerous punctures separated by less than half their diameter; metepisternum very narrow, with several deep punctures.

***First visible abdominal sternite***: disk smooth, completely striate laterally.

***Legs***: protibia on outer margin with seven or eight low teeth topped by short denticle, in-between teeth minute denticles appear, inserted directly on outer protibial margin; protarsal groove deep, straight. Protibial spur conspicuous, inserted near tarsal insertion, another shorter and stouter spur inserted on anterior protibial margin; protarsus well developed; protarsal claws strong, bent. Mesotibia and metatibia slightly widened, with two rows of short sparse denticles inserted along outer margin. Meso- and metatarsus rather short and thickened.

**Figure 12.**
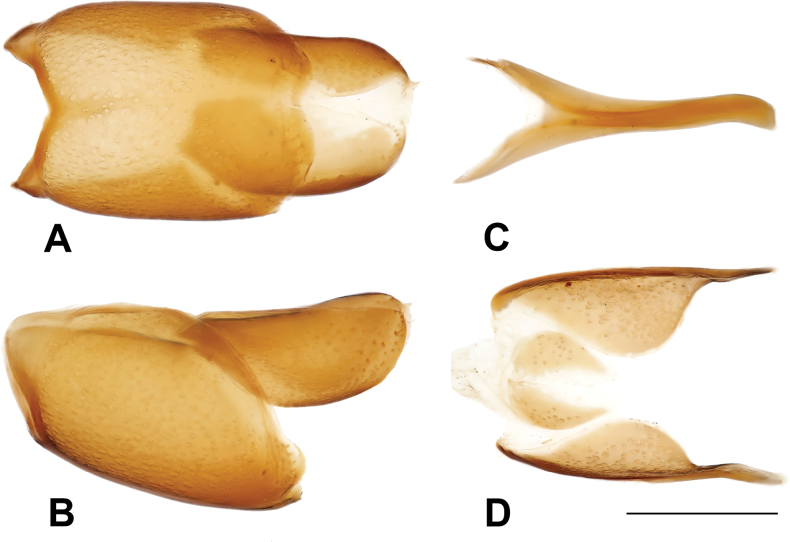
Margarinotus (Grammostethus) birmanus Lundgren, 1992 (= *Hister gentilis* Lewis, 1891), paralectotype, male terminalia. **A**. 8^th^ tergite and sternite, ventral view; **B**. Ditto, lateral view; **C**. 9^th^ sternite, ventral view; **D**. 9^th^ and 10^th^ tergites, dorsal view. Scale bar: 0.5 mm.

***Male genitalia***: Aedeagus (Fig. 1A–C) thickest in middle, gradually tapering apically. Translucent ring near tegmen base present (this condition is similar to M. (G.) mazuri sp. nov.). Tergites and sternites VIII–X as in Fig. 12.

##### Distribution.

Myanmar, Laos. Occurrence in mainland China possible. Described from Myanmar (= Burma), later reported from Taiwan (Mazur 2007). We have seen photographs of Taiwanese specimens identified as M. (G.) birmanus by Mazur and we believe they represent a different species. Its occurrence in Taiwan therefore remains questionable. Mazur (2013) reported this species as new to Laos; Gomy and Vienna (2013) reported it later that year from that country as well. We were able to examine the specimen mentioned by Mazur and list it here as *M.* (*G.*) c.f. birmanus (since its dorsal elytral stria IV is intermittent, see above); the couple mentioned by Gomy and Vienna (2013) was also examined and corresponds well to the examined syntypes.

##### Biology.

No biological data available.

##### Remarks.

**Figure 13.**
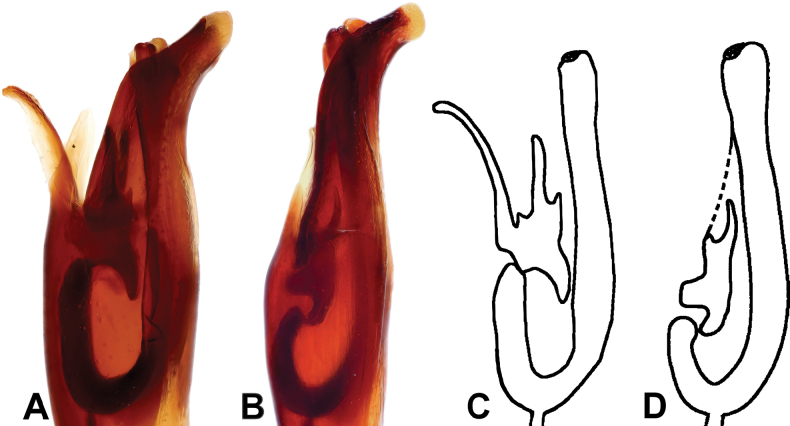
Apico-lateral view of adeagi and comparison of median armatures between putatively closely related species. Margarinotus (Grammostethus) birmanus (**A, C**) Margarinotus (Grammostethus) mazuri sp. nov. (**B, D**).

M. (Grammostethus) mazuri sp. nov. is very similar to M. (G.) birmanus, including the shared translucent circle at base of tegmen. Main differences are: 1) punctures on propygidium and pygidium of M. (Grammostethus) mazuri sp. nov. are significantly denser and coarser than those of M. (G.) birmanus, 2) dorsal elytral striae of M. (Grammostethus) mazuri sp. nov. are more impressed than those of M. (G.) birmanus, 3) median armatures of aedeagi differ as well (Fig. 13).

###### Subgenus *Ptomister* Houlbert & Monnot, 1922

#### Margarinotus (Ptomister) deficiens sp. nov.

Taxon classificationAnimaliaColeopteraHisteridae

05EB030E-8E7C-5173-A27E-20C946DF9D32

https://zoobank.org/E5A644C8-05E7-4E61-9635-4EC0AD94A239

Figs 14, 15, 16

##### Type material.

***Holotype***: China • ♂, glued on a card, with genitalia in a separate microvial, with following labels: “广东省肇庆黑石顶,2013-X-4~6,贾凤龙 贾粤 陈冰捷 林仁超 徐伟林 采” [= China, Guangdong, Zhaoqing, Heishiding Nature Reserve. 4–6 Oct 2013. Fenglong JIA, Yue JIA, Bingjie CHEN, Renchao LIN, Weilin XU leg.] [printed], followed by: “SYSU En-423481” [printed], followed by: “HOLOTYPE” [red label, printed] (SYSU). ***Paratypes***: China • 1 specimen, glued on a card, with genitalia in a separate microvial, with following labels: “广东省肇庆黑石顶,2013-X-4~6,贾凤龙 贾粤 陈冰捷 林仁超 徐伟林 采” [= China, Guangdong, Zhaoqing, Heishiding Nature Reserve. 4–6 Oct 2013. Fenglong JIA, Yue JIA, Bingjie CHEN, Renchao LIN, Weilin XU leg.] [printed], followed by: “SYSU En-423482” [printed], followed by: “PARATYPE” [yellow label, printed] (SYSU). China • 1 specimen, idem as proceeding but “SYSU En-423483” (SYSU). China • 1 specimen, idem as proceeding but “SYSU En-423484” (GIZ). China • 1 specimen, idem as proceeding but “SYSU En-423485” (CJZH). • Paratype, male, side-mounted on mounting card, right elytron pierced, right protarsus broken off, genitalia extracted and glued to the same card as the specimen, with following labels: “广西那坡弄化” [= Nonghua Village, Napo County, Guangxi Zhuang Autonomous Region, China] / 960m / 1998.IV.13 周海生 [= Haisheng ZHOU leg / 中科院动物所 [= Institute of Zoology, Chinese Academy of Sciences, Beijing, China] (black-framed, printed label), followed by: “Margarinotus / sp. nov. / Identified by (in Chinese): S. Mazur” (black-framed, printed-written label), followed by red, printed paratype label: “PARATYPE / *Margarinotus* / (*Ptomister*) deficiens / sp. nov. Det. J. Zheng / & T. Lackner 2025” (IZ-CAS). • Paratype, male, side-mounted on a card, right elytron pierced, torn from body and glued to the same mounting card as specimen, last two tarsomeres of left mesotarsus broken off, genitalia extracted, glued to the same mounting card as specimen, with following labels: “广西那坡弄化” [= Nonghua Village, Napo County, Guangxi Zhuang Autonomous Region, China] / 960m / 1998.IV.13 周海生 [= Haisheng ZHOU leg / 中科院动物所 [= Institute of Zoology, Chinese Academy of Sciences, Beijing, China] (black-framed, printed label), followed by red, printed paratype label. “PARATYPE / *Margarinotus* / (*Ptomister*) deficiens / sp. nov. Det. J. Zheng / & T. Lackner 2025” (CTLA). • Paratype, female, side-mounted on a card, right elytron pierced, left lateral metaventral cuticular disk torn from body, glued to the same mounting card as specimen, with labels identical to male paratype, with additional red label: “PARATYPE / *Margarinotus* / (*Ptomister*) deficiens / sp. nov. Det. J. Zheng / & T. Lackner 2025” (IZ-CAS).

##### Differential diagnosis.

This species is, due to its multidentate protibia, very similar to M. (Ptomister) multidens (Schmidt, 1889), which occurs likewise in Central and Southern China (prov. Sichuan, Fujian) and in Taiwan, Myanmar, and northern India (Mazur 2011). *Margarinotus* (*Pt.*) deficiens sp. nov., however, is easily distinguished from it by absent frontal and supraorbital striae, and by a strongly shortened outer lateral pronotal stria, which is present as a mere apical fragment in anterior angles. The outer lateral stria of M. (Ptomister) multidens is well impressed and complete.

##### Description.

***Body length***: 5.42–6.31 mm (PEL: average = 5.03, *n* = 5); width: 3.88–4.85 mm (EW: average = 4.32, *n* = 5). Biometric data given in Table 1. Body oval, black, shiny; body appendages dark brown.

**Figure 14.**
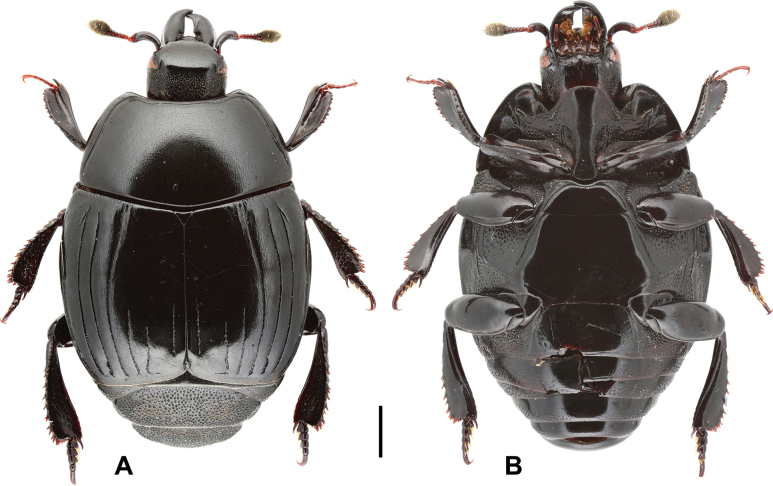
Margarinotus (Ptomister) deficiens sp. nov., habitus. **A**. Dorsal view; **B**. Ventral view. Scale bar: 1.0 mm.

***Head***: frons (Fig. 14) flat; frontal disk microscopically punctate, almost glabrous; frontal stria absent, slightly impressed behind eyes; supraorbital and occipital striae absent. Mandibles finely and sparsely microscopically punctate; eyes flattened, visible from above; labrum almost glabrous, finely punctate.

***Pronotum***: marginal pronotal stria (Fig. 14) broadly interrupted behind head, complete laterally. Inner lateral pronotal stria complete, deeply impressed, with feeble angulation behind eyes; outer lateral pronotal stria occasionally present as intermittent microscopic fragment in anterior pronotal corners. Pronotal disk almost glabrous, with scattered microscopic punctures; pronotal hypomeron glabrous.

***Elytra***: elytral disk glabrous, faint microscopic punctures appear only along apices of external subhumeral stria. Elytral epipleuron densely and coarsely punctate; marginal elytral stria absent. Marginal epipleural stria complete, with coarse punctures. External subhumeral as well as dorsal elytral striae I–III complete; apical third interspersed with punctures. Dorsal elytral stria IV and V represented as short punctate apical fragments; stria IV runs slightly further basally than stria V. Sutural elytral stria abbreviated slightly from elytral apex, interspersed with punctures, present on apical third, running slightly longer than stria IV basally. Oblique humeral stria present on basal third, very fine (Fig. 14).

***Abdomen***: propygidium with an indistinct depression postero-laterally, densely and deeply punctured; punctures separated by less than their diameter, intermingled with minute punctures. Punctures of pygidium similar to those on propygidium, albeit coarser and denser.

***Prosternum***: prosternal lobe rounded apically, marginal stria laterally deeply impressed, antero-medially evanescent. Disk of prosternal lobe postero-laterally with large and deep punctures, disappearing antero-mediad; rest of disk with scattered fine microscopic punctation. Prosternal keel almost glabrous; carinal prosternal striae absent; laterally disk coarsely punctate; lateral prosternal striae cariniform, almost straight.

***Mesoventrite***: anterior margin of mesoventrite feebly emarginate medially; marginal mesoventral stria complete and subcariniform; fragments of complimentary short stria present behind each anterior angle. Meso-metaventral suture complete, subcariniform; mesoventral disk smooth.

***Metaventrite***: lateral metaventral stria bisinuate, carinate, extending obliquely and posteriorly, united with oblique stria. Post-mesocoxal stria extending along posterior margin of mesocoxa, its outer end attaining middle of metaventral-mesepimeral suture. Intercoxal disk of metaventrite finely and sparsely punctate (Fig. 14), almost smooth; lateral disk of metaventrite densely covered with large setigerous punctures on basal half, becoming sparser and smaller on apical half. Intercoxal disk of first visible abdominal sternite completely striate laterally, smooth.

***Legs***: protibia with ~ 12 small denticles in two tightly-set rows on outer margin; mesotibia slightly dilated, outer margin with three different rows of denticles, of which the denticles in middle row the stoutest, densely abutting each other apically; mesotibial spur well-developed, stout, its length ~ 1.5 × as first mesotarsomere; metatibia in all aspects similar to mesotibia.

**Figure 15.**
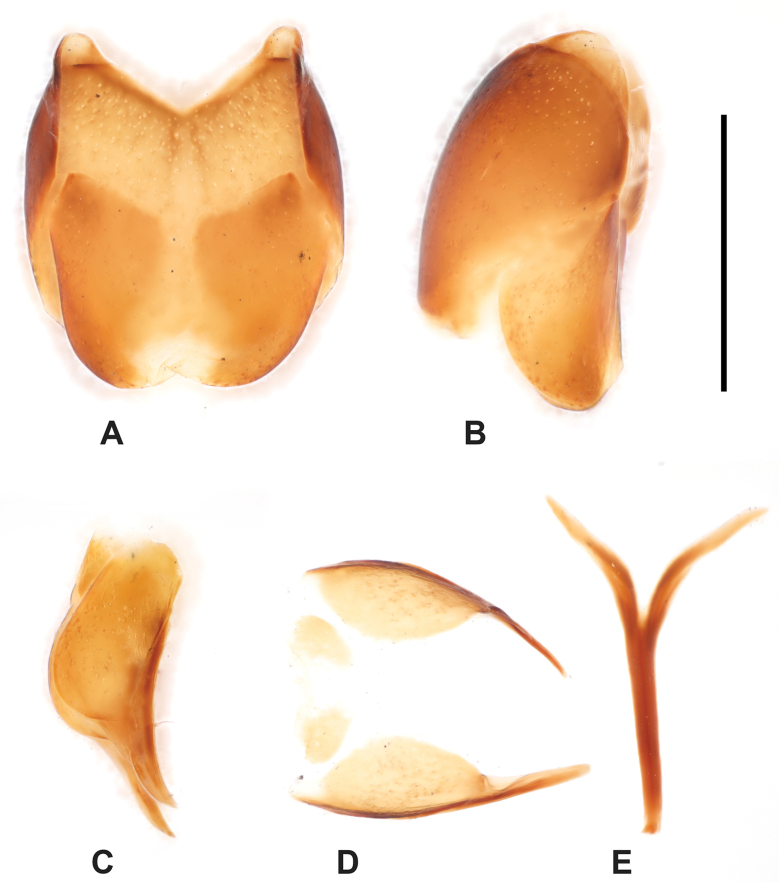
Margarinotus (Ptomister) deficiens sp. nov., male terminalia. **A**. 8^th^ tergite and sternite, ventral view; **B**. Ditto, lateral view; **C**. 9^th^ and 10^th^ tergites, lateral view; **D**. Ditto, dorsal view; **E**. 9^th^ sternite, ventral view. Scale bar: 1.0 mm.

**Figure 16.**
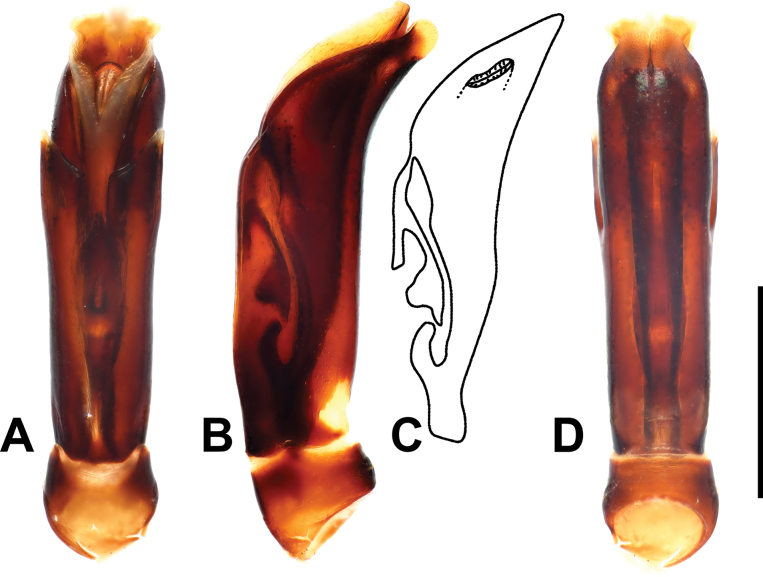
Margarinotus (Ptomister) deficiens sp. nov., aedeagus. **A**. Dorsal view; **B**. Lateral view; **C**. Median armature; **D**. Aedeagus, ventral view. Scale bar: 1.0 mm.

***Male genitalia***: aedeagus: basal piece very short, ratio in length of parameres to basal piece ~ 1:6; tegmen short and stout, rather thick; median armature well visible from ventral view (Fig. 16). Tergites and sternites VIII–X on Fig. 15.

##### Distribution.

China (Guangdong, Guangxi Zhuang Autonomous Region).

##### Biology.

Specimens were collected in rotten bananas (cf. *Musa basjoo*)​.

##### Etymology.

The specific epithet refers to the absence of frontal and supraorbital striae, a unique feature for the genus.

##### Remarks.

This newly described species represents the only known *Margarinotus* completely lacking frontal and supraorbital striae. Its outer lateral pronotal stria is rudimentary, present only as apical fragments in anterior angles.

### Updated keys to *Margarinotus* Marseul, 1854 of China

Zhou et al. (2022) published a comprehensive key to *Margarinotus* species occurring in China in both English and Chinese. Their key mentioned omitting several species, mostly occurring in the Taiwan region of China, or Japan and Korea. The omitted taxa were Margarinotus (Asterister) curvicollis (Bickhardt, 1913) (Taiwanese endemic), Margarinotus (Grammostethus) arrosor (Bickhardt, 1920), comb. nov. (present paper), Margarinotus (Ptomister) sutus (Lewis, 1884) (known from Japan and Korea), Margarinotus (Grammostethus) birmanus Lundgren, 1992 (known from Myanmar, Laos, and doubtfully also from the Taiwan region of China, present paper) and Margarinotus (Grammostethus) taiwanus Mazur, 2008 (Taiwanese endemic). Here, we present a revised key including all above-mentioned species, which we also illustrate with high-quality photographs. Apart from M. (G.) arrosor and M. (G.) birmanus (treated here), all species have recently been either fully described or re-described (see e.g. Ôhara 1989, 1999). For the sake of completeness we provide a re-description of M. (G.) birmanus in our contribution, as we believe it may be recorded from mainland China in future.

#### Keys to *Margarinotus* Marseul, 1854 of China

**Table:** 

**Key to subgenera**
1(2)	Sutural elytral stria complete, basally connected with basal fragment of elytral stria V; legs, antennae, mouthparts, lateral areas of propygidium + pygidium reddish-brown	**subgenus *Asterister* Desbordes, 1920**
2(1)	Sutural elytral stria usually not complete, abbreviated basally, usually basally not connected with basal fragment of elytral stria V; body completely black, without reddish-brown parts	**3**
3(8)	Pronotum (at least) in apical angles with three striae: marginal pronotal stria and two lateral pronotal striae – outer and inner, usually both lateral pronotal striae complete. Aedeagus occasionally with apically “open” un-fused parameres with eversible median lobe (Figs 4, 8)	**4**
4(5)	Meso-and metatibiae broadened, triangular, covered with yellow pubescence; pronotal hypomeron with very short setae	**subgenus *Eucalohister* Reitter, 1909**
5(4)	Meso-and metatibiae usually not broadened, “normal”, in most cases without yellow pubescence; pronotal hypomeron glabrous	**6**
6(7)	Aedeagus with apically “open” un-fused parameres with eversible median lobe (Figs 4, 8); prosternal apophysis usually with two striae; anterior margin of mesoventrite medially only slightly emarginate, almost straight	**subgenus *Grammostethus* Lewis, 1906 (part)**
7(6)	Aedeagus apically fused, without eversible median lobe (Fig. 16); prosternal apophysis usually without striae; anterior margin of mesoventrite usually deeply emarginate medially	**subgenus *Ptomister* Houlbert & Monnot, 1922**
8(3)	Pronotum in apical angles with two striae: marginal pronotal stria and a single lateral pronotal stria. Aedeagus occasionally with apically “open” un-fused parameres with eversible median lobe (Figs 4, 8)	**9**
9(10)	Aedeagus with apically “open” un-fused parameres with eversible median lobe (Figs 4, 8)	**subgenus *Grammostethus* Lewis, 1906 (part)**
10(9)	Aedeagus without apically “open” un-fused parameres with eversible median lobe	**11**
11(12)	Marginal pronotal stria usually complete or almost complete; body subcylindrical; tibiae thickened; metafemur widened	**subgenus *Stenister* Reichardt, 1926**
12(11)	Marginal pronotal stria shortened basally, usually reaching pronotal mid-length; body roundly oval or ovoid; tibiae not thickened, metafemur “normal”	**subgenus *Paralister* Bickhardt, 1917**
**Subgenus *Asterister* Desbordes, 1920**
This subgenus has not yet been documented from mainland China. From the island of Taiwan, a single endemic species M. (A.) curvicollis (Bickhardt, 1913) (Fig. 17) has been recorded; its presence on mainland China is probable.
**Subgenus *Eucalohister* Reitter, 1909**
1	Dorsal elytral stria V absent	**Margarinotus (Eucalohister) bipustulatus (Schrank, 1781)** [westernmost China: Xinjiang, Kazakhstan, Turkmenistan, Russia (Siberia), Ukraine, Caucasus Mountains, Europe]
2(1)	Dorsal elytral stria V present on apical fourth	**Margarinotus (Eucalohister) gratiosus (Mannerheim, 1852)** [China: northeast China, Inner Mongolia, Qinghai, Ningxia, Mongolia, Russia (eastern Siberia)]
**Subgenus *Grammostethus* Lewis, 1906**
1(6)	Pronotum with two lateral pronotal striae	**2**
2(3)	Dorsal elytral striae I-III complete; stria IV vaguely impressed, intermittent; pygidia with fine scattered punctures; pygidial apex glabrous	**Margarinotus (Grammostethus) arrosor (Bickhardt, 1920), comb. nov**. [southern China: Fujian, Hong-Kong, Guangdong]
3(2)	Dorsal elytral striae I-IV complete	**4**
4(5)	Body roundly-oval, cuticle black; appendages dark brown; pygidia with finer scattered punctures separated by 1–3 × their diameter	**Margarinotus (Grammostethus) birmanus Lundgren, 1992** [China: Taiwan (?), Myanmar, Laos]
5(4)	Body elongate-oval, cuticle dark brown; appendages reddish-brown; pygidia with large dense and coarse punctures separated by 0.5–1 × their diameter	**Margarinotus (Grammostethus) mazuri sp. nov**. [China: Guangdong, Jiangxi]
6(1)	Pronotum with a single lateral pronotal stria	**7**
7(8)	Protibia with enlarged apical-most tooth, topped by three denticles (for fig. see Mazur 2008: fig. 5) followed by three low teeth each topped by tiny denticle	**Margarinotus (Grammostethus) taiwanus Mazur, 2008 (Fig. 18)** [China: Taiwan]
8(7)	Protibia multidentate; apical-most tooth not enlarged	**9**
9(10)	Dorsal elytral striae I-III complete; stria IV present only as short apical fragment; lateral pronotal stria distinctly distanced from pronotal margin	**Margarinotus (Grammostethus) schneideri Kapler, 1996** [China: Beijing, Gansu, Sichuan]
10(9)	Dorsal elytral striae I-IV complete	**11**
11(12)	Inner area of lateral pronotal stria with a broad band of coarse punctures	**Margarinotus (Grammostethus) occidentalis (Lewis, 1885)** [China: Fujian, Guangdong, Guizhou, Shanghai]
12(11)	Inner area of lateral pronotal stria without punctures	**13**
13(14)	Lateral metaventral stria apically joining oblique metaventral stria	**Margarinotus (Grammostethus) impiger (Lewis, 1905)** [China: Yunnan]
14(13)	Lateral metaventral stria apically not joining oblique metaventral stria	**15**
15(16)	Marginal stria of prosternal lobe complete	**Margarinotus (Grammostethus) niponicus (Lewis, 1885)** [China (Beijing, Hebei, Jiangxi, Hubei, Sichuan, Shaanxi, Taiwan), Japan, Korea, Russia (Far East)]
16(15)	Marginal stria of prosternal lobe interrupted medially	**17**
17(18)	Dorsal elytral stria V and sutural elytral stria shorter, not reaching elytral mid-length basally; carinal prosternal striae indistinct or absent	**Margarinotus (Grammostethus) formosanus Ôhara, 1999** [China: Taiwan]
18(17)	Dorsal elytral striae V and sutural stria longer, surpassing elytral mid-length basally; carinal prosternal striae distinct	**19**
19(20)	Carinal prosternal striae basally not united	**Margarinotus (Grammostethus) fragosus (Lewis, 1892)** [China: Yunnan, Myanmar]
20(19)	Carinal prosternal striae united basally	**Margarinotus (Grammostethus) stercoriger (Marseul, 1880)** [China: Yunnan, Indonesia: Sumatra, Vietnam]
**Subgenus *Ptomister* Houlbert & Monnot, 1922**
1(4)	Carinal prosternal striae present (Fig. 19)	**2**
2(3)	Dorsal elytral striae I-III complete	**Margarinotus (Ptomister) babai Ôhara, 1999** [China: Taiwan]
3(2)	Dorsal elytral striae I-IV complete	**Margarinotus (Ptomister) sutus (Lewis, 1884) (Fig. 19)** [China: Liaoning, Japan, Korea]
4(1)	Carinal prosternal striae absent	**5**
5(6)	Frontal and supraorbital striae absent (Fig. 14); inner lateral pronotal stria present as minute fragments in apical pronotal corners	**Margarinotus (Ptomister) deficiens sp. nov**. [China: Guangdong, Guangxi Zhuang Autonomous Region]
6(5)	Frontal and supraorbital striae present. Inner lateral pronotal stria normally developed	**7**
7(16)	Dorsal elytral striae I-III complete; stria IV variously abbreviated basally	**8**
8(9)	Inner lateral pronotal stria strongly undulate behind eyes	**Margarinotus (Ptomister) boleti (Lewis, 1884)** [China: Taiwan, Japan, Russia: Kuril Islands]
9(8)	Inner lateral pronotal stria normally developed, straight, not strongly undulate behind eyes	**10**
10(11)	Outer lateral pronotal stria complete	**Margarinotus (Ptomister) koltzei (Schmidt, 1889)** [China: Heilongjiang, Japan, Russian Far East]
11(10)	Outer lateral pronotal stria usually basally shortened or interrupted medially	**12**
12(15)	Dorsal elytral stria IV shortened, not surpassing elytral mid-length basally; stria V vaguely impressed, indistinct	**13**
13(14)	Outer lateral pronotal stria present on apical half or slightly surpassing it	**Margarinotus (Ptomister) incognitus (Marseul, 1854)** [China: Shaanxi, Sichuan, Taiwan, Yunnan, Northern India, Nepal]
14(13)	Outer lateral pronotal stria complete, occasionally medially interrupted	**Margarinotus (Ptomister) tristriatus Wenzel, 1944** [China: Heilongjiang, Beijing, Russia: Eastern Siberia, Far East]
15(12)	Dorsal elytral stria IV surpassing elytral half basally, shortened on basal 1/6 to 1/3; stria V well impressed, distinct	**Margarinotus (Ptomister) striola striola (C.R. Sahlberg, 1819)** [China: Heilongjiang, Jilin, Northern Scandinavia, Northern Europe, Russia: Eastern Siberia, Far East]
16(7)	Dorsal elytral striae I-IV complete	**17**
17(20)	Oblique stria of metaventrite indistinct	**18**
18(19)	Protibia with multiple small denticles on outer margin; dorsal elytral stria V with a short basal rudiment	**Margarinotus (Ptomister) multidens (Schmidt, 1889)** [China: Chongqing, Fujian, Guangxi, Hubei, Jiangxi, Sichuan, Taiwan, Eastern India, Myanmar]
19(18)	Protibia with five teeth topped by a small denticle on outer margin; dorsal elytral stria V without a short basal rudiment	**Margarinotus (Ptomister) hailar Wenzel, 1944** [China: Heilongjiang, Inner Mongolia, Mongolia, Eastern Russia: Siberia]
20(17)	Oblique stria of metaventrite distinct	**21**
21(26)	Lateral metaventral and oblique metaventral striae united	**22**
22(23)	Lateral metaventral disk with large setigerous punctures	**Margarinotus (Ptomister) cadavericola (Bickhardt, 1920)** [China: Fujian, Heilongjiang, Liaoning, Sichuan, Japan, Russia: Far East, Kuril Islands]
23(22)	Punctures of lateral metaventral disk not setigerous	**24**
24(25)	Sutural elytral striae well impressed	**Margarinotus (Ptomister) agnatus (Lewis, 1884)** [China: Heilongjiang, Zhejiang, Japan, Northern India, Korea, Russian Far East, Sakhalin Peninsula]
25(24)	Sutural elytral striae weakly impressed, as a dotted weak line	**Margarinotus (Ptomister) osawai Ôhara, 1999** [China: Taiwan]
26(21)	Lateral metaventral stria and oblique metaventral stria not united	**27**
27(28)	Punctures of lateral disk of metaventrite setigerous	**Margarinotus (Ptomister) weymari Wenzel, 1944** [China: Beijing, Heilongjiang, Jilin, Liaoning, Japan, Korea, Russia: Far East]
28(27)	Punctures of lateral metaventral disk not setigerous	**29**
29(30)	Marginal stria of prosternal lobe interrupted medially; protibia with 8–9 teeth on outer margin, larger species (5.50–6.10 mm); prosternal apophysis occasionally with vestiges of carinal prosternal striae	**Margarinotus (Ptomister) reichardti Kryzhanovskij, 1976** [China: Beijing, Heilongjiang, Jilin, Liaoning, Taiwan, Japan, Korea, Russia: Far East]
30(29)	Marginal stria of prosternal lobe uninterrupted; protibia with 5–6 teeth on outer margin, smaller species (3.50–3.80 mm); prosternal apophysis never with vestiges of carinal prosternal striae	**Margarinotus (Ptomister) wenzelianus Kryzhanovskij, 1976** [China: Heilongjiang, Liaoning, Mongolia, Russia: Far East]
**Subgenus *Stenister* Reichardt, 1926**
Only a single species, Margarinotus (Stenister) obscurus (Kugelann, 1792) has so far been documented from westernmost China (Xinjiang). Widely distributed in western Palaearctic.
**Subgenus *Paralister* Bickhardt, 1917**
1(6)	Dorsal elytral striae I-III complete; stria IV always basally shortened.
2(3)	Marginal stria of prosternal lobe complete; marginal mesoventral stria abbreviated on posterior third	**Margarinotus (Paralister) oblongulus (Schmidt, 1892)** [China: Xinjiang, Kyrgyzstan, Uzbekistan]
3(2)	Marginal stria of prosternal lobe present only on apical half or outright absent; marginal mesoventral stria nearly complete	**4**
4(5)	Prosternal process narrow and convex	**Margarinotus (Paralister) laevifossa (Schmidt, 1890)** [China: Xinjiang, Kyrgyzstan, Turkmenistan]
5(4)	Prosternal process broad and flattened	**Margarinotus (Paralister) periphaerus Mazur, 2003** [China: Beijing, Gansu, Henan, Ningxia, Shaanxi, Shanxi]
6(1)	Dorsal elytral striae I-IV complete	**7**
7(8)	Each elytron with vaguely formed reddish macula; elytral epipleuron impunctate	**Margarinotus (Paralister) purpurascens (Herbst, 1792)** [China: Xinjiang, Uzbekistan, Russia: Far East, Siberia, Korean Peninsula, Caucasus Mountains, Europe]
8(7)	Elytra black; elytral epipleuron punctate	**Margarinotus (Paralister) koenigi (Schmidt, 1888)** [China: Heilongjiang, Jilin, Korea, Mongolia, Russia: Amurskij Kray]

## Discussion

Wenzel (1944) conducted a comprehensive study of the genus *Margarinotus*, incorporating within it taxa *Paralister*, *Stenister*, *Grammostethus*, and a substantial portion of the genus *Hister*, based primarily on similarities in genitalic morphology. This taxonomic revision stimulated considerable discourse and subsequent investigations (e.g., Kryzhanovskij 1966; Mazur 1972; Kryzhanovskij and Reichardt 1976; Vienna 1977; Olexa 1982; Ôhara 1989). Although the genus is currently subdivided into ten recognized subgenera, no phylogenetic analyses have yet been published to assess the validity of these subgeneric divisions. To date, species assignment within subgenera has relied predominantly on morphological characters and their combinations. Ôhara (1989) provided a discussion on the potential phylogenetic significance of genitalic traits within *Margarinotus*, though his study lacked formal analytical methodology and yielded inconclusive results. For classification of Holarctic *Margarinotus* species into subgenera, keys provided by Kryzhanovskij and Reichardt (1976) and Zhou et al. (2022) remain practical and widely employed. However, in attempting to classify newly described species from southern China, we encountered difficulties that prompted extensive dialogue within the histeridological community.

**Figure 17.**
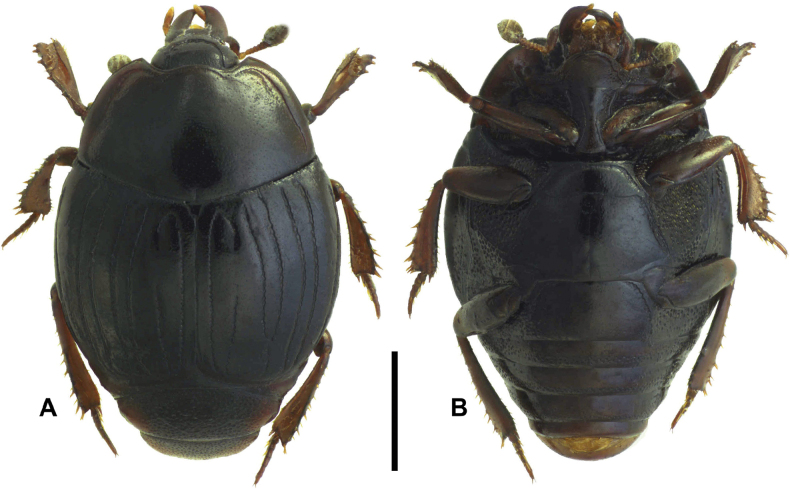
Margarinotus (Asterister) curvicollis (Bickhardt, 1913), habitus. **A**. Dorsal view; **B**. Ventral view. Scale bar: 1.0 mm.

**Figure 18.**
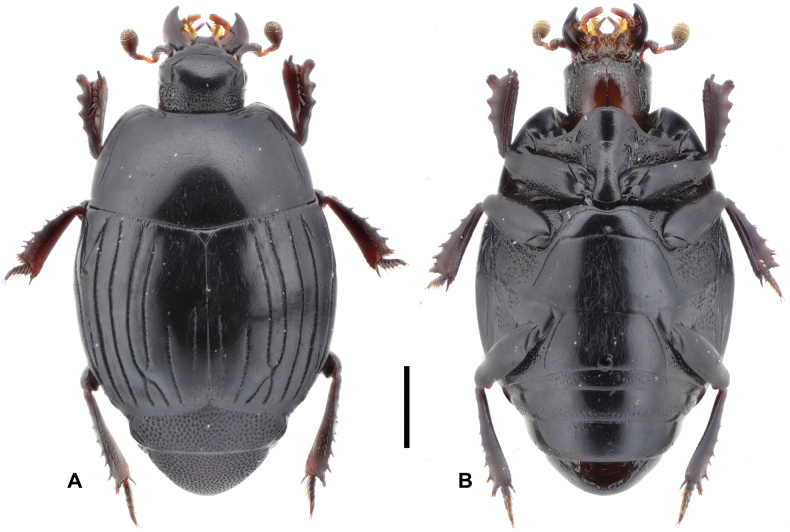
Margarinotus (Grammostethus) taiwanus Mazur, 2008, habitus. **A**. Dorsal view; **B**. Ventral view. Scale bar: 1.0 mm.

**Figure 19.**
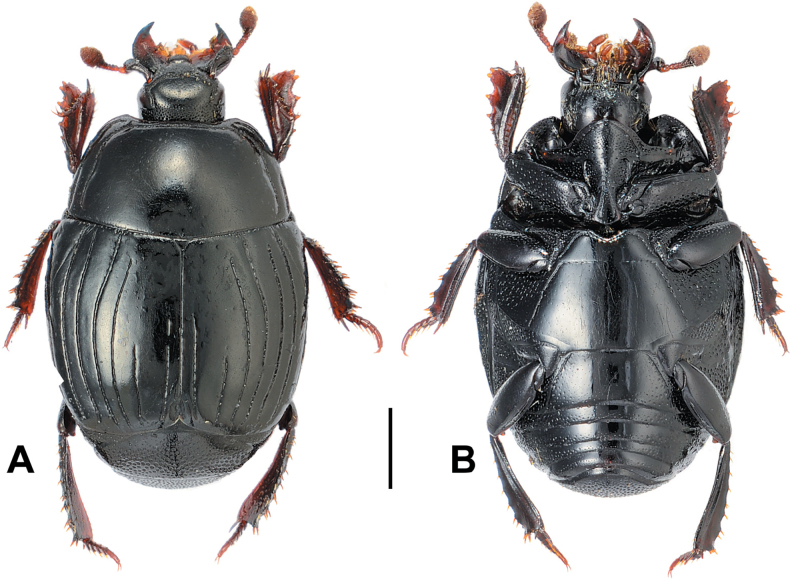
Margarinotus (Ptomister) sutus (Lewis, 1884), habitus. **A**. Dorsal view; **B**. Ventral view. Scale bar: 1.0 mm.

Following considerable discussion and consultation with leading specialists, we opted to follow the guidance of Professors Masahiro Ôhara (Sapporo, Japan) and Sławomir Mazur (Warsaw, Poland) in assigning two of our herein included species, *M. mazuri* and *M. arrosor*, to the subgenus *Grammostethus*, despite their possession of both inner and outer lateral pronotal striae. According to these experts, the most diagnostically reliable character for subgeneric classification within *Margarinotus* is the morphology of the aedeagus. In *Grammostethus*, this is characterized by an eversible, “open” apical portion of parameres comprising ~ 1/3 of the aedeagus length, as illustrated by Ôhara (1999: fig. 3A–C). Both *M. mazuri* and *M. arrosor* exhibit this genitalic feature, justifying their placement in *Grammostethus*.

Conversely, members of the subgenus *Ptomister* are distinguished by the presence of both lateral pronotal striae and apically swollen aedeagus with fused parameres lacking an eversible apical third (see Ôhara 1999: fig. 12A, B). Our newly described species *M. deficiens* does not entirely conform to the morphological definition of *Ptomister*, as its outer lateral pronotal stria is almost absent, present only as short fragments at the anterior corners. Nevertheless, the structure of its aedeagus is consistent with that of *Ptomister*, warranting its inclusion in this subgenus. We emphasize that the subgeneric placements proposed herein are provisional. We strongly advocate for a comprehensive phylogenetic analysis of *Margarinotus*, employing an integrative approach that combines both morphological and molecular data to achieve a more robust taxonomic framework.

As of now, a total of 34 species within genus *Margarinotus* has been recorded in China (Zhou et al. 2022). However, *Margarinotus* distribution in Guangdong and Jiangxi Provinces is poorly recorded; only Margarinotus (Grammostethus) occidentalis (Lewis, 1885) has been documented from Guangdong (Zhou et al. 2022), while a mere two species, Margarinotus (Ptomister) multidens (Schmidt, 1889) and Margarinotus (Grammostethus) niponicus (Lewis, 1895) are known from Jiangxi (Zhou et al. 2022). Zhou et al. (2022) mention Margarinotus (Ptomister) multidens (Schmidt, 1889) from Guangxi. Interestingly, the two newly described species and Margarinotus (Grammostethus) arrosor (Bickhardt, 1920) are all distributed in Fujian Province, which is adjacent to the provinces of Guangdong and Jiangxi. The two newly described species expand the known distribution of *Margarinotus* in Guangdong, Guangxi Zhuang Autonomous Region, and Jiangxi.

## Supplementary Material

XML Treatment for Margarinotus (Grammostethus) mazuri

XML Treatment for Margarinotus (Grammostethus) arrosor

XML Treatment for Margarinotus (Grammostethus) birmanus

XML Treatment for Margarinotus (Ptomister) deficiens

## Additional information

### Use of AI

No use of AI was reported.

### Author ORCIDs

Haidong Yang https://orcid.org/0000-0002-3157-2090

Tomáš Lackner https://orcid.org/0000-0002-0108-5785

### Data availability

All of the data that support the findings of this study are available in the main text.
